# Combinations of Symptoms in Emergency Presentations: Prevalence and Outcome

**DOI:** 10.3390/jcm8030345

**Published:** 2019-03-12

**Authors:** Tobias Kuster, Christian H. Nickel, Mirjam A. Jenny, Lana L. Blaschke, Roland Bingisser

**Affiliations:** 1 Emergency Department, University Hospital Basel, Petersgraben 2, CH-4031 Basel, Switzerland; tobias.kuster@usb.ch (T.K.); christian.nickel@usb.ch (C.H.N.); 2 Harding Center for Risk Literacy, Max Planck Institute for Human Development, Lentzeallee 94, DE-14195 Berlin, Germany; jenny@mpib-berlin.mpg.de (M.A.J.); blaschke@mpib-berlin.mpg.de (L.L.B.)

**Keywords:** emergency medicine, emergency department, symptom-oriented research, nonspecific complaints, symptoms

## Abstract

The predictive power of certain symptoms, such as dyspnoea, is well known. However, research is limited to the investigation of single chief complaints. This is in contrast to patients in the emergency department (ED) presenting usually more than one symptom. We aimed to identify the most common combinations of symptoms and to report their related outcomes: hospitalisation, admission to intensive care units, and mortality. This is a secondary analysis of a consecutive sample of all patients presenting to the ED of the University Hospital Basel over a total time course of 6 weeks. The presence of 35 predefined symptoms was systematically assessed upon presentation. A total of 3960 emergency patients (median age 51, 51.7% male) were included. Over 130 combinations of two, 80 combinations of three, and 10 combinations of four symptoms occurred 42 times or more during a total inclusion period of 42 days. Two combinations of two symptoms were predictive for in-hospital mortality: weakness and fatigue (Odds ratio (OR) = 2.45), and weakness and headache (OR = 3.01). Combinations of symptoms were frequent. Nonspecific complaints (NSCs), such as weakness and fatigue, are among the most frequently reported combinations of symptoms, and are associated with adverse outcomes. Systematically assessing symptoms may add valuable information for prognosis and may therefore influence triage, clinical work-up, and disposition.

## 1. Introduction

In the emergency department (ED), patients present with a wide range of symptoms and an even wider range of combinations of symptoms. Assessing and interpreting these symptoms plays a crucial role in triage, routine workup, preliminary clinical diagnosis, as well as treatment and disposition [1]. Recent studies have increased the knowledge of the prognostic power of certain symptoms, e.g., dyspnoea or general disability, regarding short- and long-term outcomes [2,3,4,5,6]. However, past research mostly focused on a small, physician-defined list of so-called “chief complaints” [2,4]. The primary reason, most often a symptom, leading to an ED presentation is the “chief complaint” which carries important prognostic information. Previous studies were limited to the investigation of single symptoms. These did not take into account that patients reporting more than one symptom at presentation are the norm rather than the exception [3,7], and that combinations of certain symptoms might be able to predict adverse outcomes. 

Due to the finding that the majority of patients report multiple symptoms at presentation, we aimed to identify the most common combinations of symptoms and to assess their associated outcomes.

## 2. Experimental Section

### 2.1. Study Design and Setting

This is a pre-planned secondary analysis of a prospective, consecutive all-comer study to assess the predictive power of the most prevalent symptom combinations at ED presentation regarding adverse outcomes. Parts of the methods used in this study have been previously described [3]. Data collection was performed at the ED of the University Hospital Basel, Switzerland, a tertiary care university hospital with an annual census of over 50,000 patients. Patients were included 24 h a day, 7 days a week, during two 3-week periods from 21st October to 11th November, 2013, and from 1st February to 23rd February, 2015. The study protocol was approved by the local ethics board (236/13, www.eknz.ch).

### 2.2. Study Population

All patients presenting to the ED with acute medical or surgical complaints were eligible. Paediatric, obstetric and ophthalmologic patients were not included, as they were treated in separate facilities nearby. Patients not willing or unable to participate, due to unconsciousness, intoxication, language problems, severe dementia, or ongoing acute life support, were not included. 

### 2.3. Study Protocol

Upon presentation, an electronic health record (EHR) was opened for every patient presenting to the ED. Patients were triaged according to the German version of the Emergency Severity Index (ESI) by a triage nurse [8]. A systematic interview was conducted by a member of the study team. The study team consisted of trained medical students.

Every patient was asked whether any of the following 35 predefined symptoms were present at presentation: Feeling feverish, rash, headache, dizziness, acute visual disorder, acute hearing disorder, nasal discharge, sore throat, cough, expectoration, dyspnoea, chest pain, abdominal pain, nausea, vomiting, diarrhoea, constipation, flank pain, dysuria, neck pain, back pain, arm pain, leg pain, joint pain, joint swelling, leg swelling, altered state of mind, dysesthesia, palsy, gait disorder, speech disorder, fatigue, weakness, loss of appetite, and feeling sleepy. Textbooks [9], as well as symptom-based guidelines, developed and published by the ED of the University Hospital Basel (www.emergencystandards.com), were used to draft a first list of symptoms. An expert panel consisting of senior emergency physicians discussed the symptoms included in the list. The aim was to come up with a list small enough to ask every patient whether each symptom was present or not in a short amount of time, yet broad enough to encompass most organ systems of the body. Participating patients reporting no symptoms or symptoms outside the predefined list were also included. Additionally, the patients’ subjective main reason for ED presentation was assessed, and all results were recorded using a questionnaire. The study database was matched with the EHR database according to an individual patient ID number. Further demographic information, such as age, gender, disposition (e.g., discharge, intensive care unit (ICU), general ward, geriatrics, etc.), and in-hospital mortality, was retrieved from the EHR. 

EHR data, phone calls with patients, proxies, and primary care providers (PCPs), or written communication with PCPs were used to obtain follow-up data up to one year. The official registry and insurance data were used to retrieve the date of death.

### 2.4. Predictor Variables

The 20 most common combinations of two symptoms in our cohort were used as predictor variables for statistical analysis. Due to the small group sizes for combinations of more than two symptoms, we restricted the analysis to the most common combinations of two symptoms. 

### 2.5. Outcomes

Hospitalisation, ICU admission, in-hospital mortality, and one-year mortality were the predefined outcomes. Direct disposition from the ED to any hospital ward, or another hospital was defined as hospitalisation. ICU admission was defined as any admission to a medical, surgical, or neurosurgical ICU, or to an intermediate care or stroke unit. In-hospital mortality was defined as death between presentation and discharge from the University Hospital Basel. One-year mortality was defined as death within one year after presentation.

### 2.6. Statistical Analysis

The statistical analysis was performed using R version 3.4.1 (https://www.R-project.org/). Multivariable logistic regression models, adjusted for age, sex, and ESI-triage-level, were performed to assess the effect of the predictor variables (combinations of two symptoms) on the outcome variables. No automated variable selection procedure was performed, as restriction to the investigation of the 20 most common combinations of two symptoms acted as a clinical variable selection method. Results are presented in Table 2 as odds ratios (OR) with 95% confidence intervals. *p*-values below 0.05 were considered to be significant.

## 3. Results

During the study period, 5634 patients presented to the ED. Out of 4703 patients screened by the study team, a total of 3960 were included for secondary analysis [3].

In brief, median age was 51 years (IQR = 33 to 71 years); 51.7% were men; the median number of symptoms was 2 (Range = 0–25). More than half of all patients, 2183 (55.1%), reported more than one, whereas 488 (12.3%) patients reported none of the 35 predefined symptoms. Out of these 488 patients, no more than 81 (2.0%) patients presented to the ED because of a non-symptom-related reason for presentation, while 338 (8.5%) patients named symptoms outside the predefined catalogue. Only 69 (1.7%) patients that could be interviewed did not report any symptom at the time interviewed by the study team, usually due to the fact that the symptom already disappeared prior to presentation.

**Figure 1:**
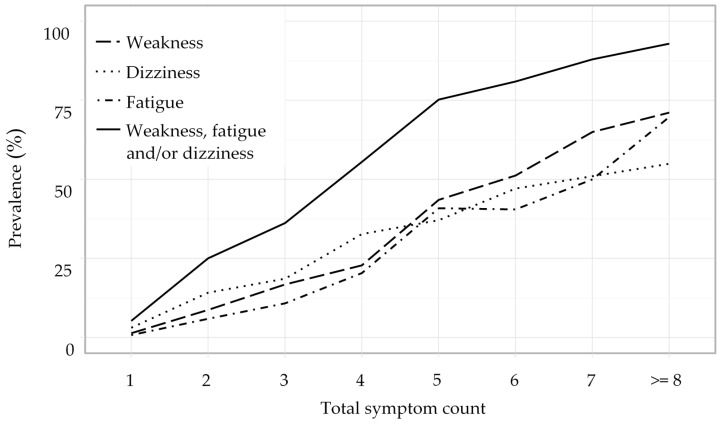
Prevalence of nonspecific complaints (NSCs) in relation to the total symptom count.

An overview over the distribution of the twenty most frequent combinations of two symptoms is given in Table 1. With 281 (7.1%) mentions, the combination of headache and dizziness was the most frequently reported. Information on the most frequent combinations of three respectively four symptoms is presented in Table A1 and Table A2 in Appendix A. Overall, over 130 combinations of two symptoms, 80 combinations of three symptoms, and 10 combinations of four symptoms occurred on average more than once per day. Nonspecific complaints (NSCs), such as weakness, dizziness, and fatigue, were frequently mentioned in combinations of two (*n* = 203, 25.1%), three (*n* = 181, 36.2%), or four symptoms (*n* = 180, 55.6%) (Figure 1). The most prevalent NSC was weakness with 556 (14%) of all patients who suffered from a wide range of underlying problems, such as falls (*n* = 48), cardiovascular (*n* = 97), respiratory (*n* = 22), infectious (*n* = 201), and neoplastic disease (*n* = 12). The effect of different combinations of two symptoms on associated outcomes is shown in Table 2. Several combinations of symptoms were predictive for hospitalisation. The combinations of weakness and headache, and fatigue and headache were predictive for ICU admission. The combinations of weakness and fatigue, and weakness and headache were predictive for in-hospital mortality. The effect of single symptoms on associated outcomes is shown in Appendix B Table A3 [3].

## 4. Discussion

This observational study on a consecutive sample of emergency patients is the first to focus on combinations of symptoms at presentation. Main results were the high prevalence of combinations of two and more symptoms and, in the case of nonspecific complaints (NSC), the combinations of three and more.

Symptoms offer relevant information on the prognosis of patients seeking ED care. This has been described for certain patient groups, e.g., patients with chest pain, dyspnoea, or stroke-like symptoms [10,11,12]. However, only a few studies on the prognostic value of different presenting complaints for short- and long-term outcomes have been conducted in all-comer populations [2,4,13]. Additionally, these studies focused on single, main, presenting, or chief complaints. Therefore, it was never taken into account that most patients present with more than one symptom [3,7]. The model of chief complaint or main symptom heavily relies on several steps of selection and reduction of information. First, the individual patients’ selection: Out of all symptoms, patients tend to choose and present the ones deemed to be most important. Only systematic interviewing may elicit all symptoms perceived at presentation. Second, the individual physicians’ selection: Out of the list of presenting symptoms, physicians tend to choose and take down the so called “chief complaint”, with a tendency to focus on frequent and specific presenting symptoms, such as chest pain, and a tendency to ignore nonspecific complaints [2,4]. 

Among the most frequent combinations of two, three and four symptoms, the high prevalence of NSCs is impressive. This finding suggests, that particularly NSCs may be filtered out by physicians [3]. This seems even more evident by comparing the reported prevalence of NSCs in studies focusing on single chief complaints [2,6,14,15,16]. Various studies have gathered data on the potential use of NSCs in the prediction of adverse outcomes, reporting a high prevalence of NSCs in older patients and an association with a higher rate of admission, increased use of resources and a longer ED-length-of-stay [5,6,15]. In a Swedish study, patients presenting to the ED with a decreased general condition were found to receive low triage priority, but had high admission rates and a three-fold increase of in-hospital death [6]. This is in accordance with our data showing a predictive power regarding short-term adverse outcomes in patients with combinations of NSCs. This knowledge seems important, as this high-risk patient group was reported to be at risk of undertriage [17,18].

In addition, females were overrepresented in the group of patients with frequent combinations of symptoms. This is most likely explained by the fact that women mention significantly more symptoms at presentation [7], and that women are more prevalent in the group of patients with NSC [19], which are overly represented in the most prevalent combinations of symptoms. 

In order to improve clinical decisions and reduce diagnostic error, machine learning approaches using vast amounts of patient data could ultimately be used, the prerequisite being unbiased and unfiltered information provided by presenting patients in high quality and high granularity.

### Limitations

Due to the single centre design of this study, results cannot be generalized. Nevertheless, the population included in this study seems to be representative for Europe, as studies in Denmark, Sweden and Germany described comparable cohorts in terms of average age, hospitalisation and ICU admission rate [2,4,14]. Second, reporting symptoms in a highly standardised, binary fashion using a list of 35 predefined symptoms carries limitations as well. However, by doing so, we limited the loss of information on the presence of symptoms as compared to routine history taking, where physicians tend to focus on typical chief complaints. We therefore consider this systematic approach to be one of the biggest strengths of the study. Third, as screening was not possible for almost 17% of all presenting patients, inclusion bias is possible. Yet, most of these patients left without being seen by a physician or were directly referred to other departments, such as eye, ear, or dermatology. Fourth, the chosen study periods may be susceptible to selection bias, as patients may present with other symptoms during other months of the year. However, we decided on these two study periods based on our experience with the case mix of patients presenting to our institution. During summer and early autumn months, we see an increase in trauma patients, whereas in December and January, presentations due to the flu and pneumonia are frequent. The chosen study periods resemble the mean patient volume presenting to our ED. Fifth, owing to the low mortality, only the most frequent combinations could be associated with patients’ outcomes. Lastly, due to the exploratory design of this study, no correction of the significance level for multiple comparisons has been made.

## 5. Conclusions

Combinations of symptoms at ED presentation are frequent and may be used to improve clinical outcome prediction. NSCs are frequent in combinations of three and four symptoms, and are highly associated with adverse outcomes. Future studies should investigate to which extent systematic assessment of symptoms could improve risk stratification tools and ultimately clinical practice.

## Figures and Tables

**Table 1:** Distribution of the 20 most frequent combinations of two symptoms.

Combination of Symptoms		Prevalence		Age	Male Sex	No. of Symptoms	ESI
Symptom 1	Symptom 2	*n*	(%)	Median (year)	(%)	Median	Median
Headache	Dizziness	281	(7.1)	45	40.9	5	3
Fatigue	Weakness	275	(6.9)	53	37.1	6	3
Weakness	Dizziness	200	(5.1)	51.5	36.5	6	3
Dizziness	Nausea	200	(5.1)	46.5	35.5	5	3
Headache	Weakness	184	(4.6)	47	38.6	7	3
Vomiting	Nausea	182	(4.6)	47	40.1	5	3
Leg pain	Joint pain	167	(4.2)	52	53.3	3	4
Cough	Headache	163	(4.1)	49	39.3	7	3
Headache	Fatigue	162	(4.1)	43.5	35.8	7	3
Fever	Cough	160	(4.0)	52.5	48.8	6	3
Fatigue	Dizziness	158	(4.0)	52.5	38.6	7	3
Dyspnoea	Chest pain	156	(3.9)	57	53.2	4	2
Headache	Nausea	153	(3.9)	44	34.6	6	3
Abdominal pain	Nausea	152	(3.8)	44	43.4	5	3
Weakness	Nausea	152	(3.8)	49.5	37.5	7	3
Fever	Headache	147	(3.7)	43	43.5	7	3
Dyspnoea	Cough	126	(3.2)	62.5	49.2	5	2
Loss of appetite	Weakness	124	(3.1)	50	33.9	7	3
Fever	Weakness	121	(3.1)	51	42.1	7	3
Cough	Weakness	121	(3.1)	53	43.8	7	3

ESI = Emergency Severity Index.

**Table 2:** Age-, sex-, and ESI-triage-level-adjusted multivariable logistic regression analysis.

Combination of Symptoms			Hospitalization		ICU Admission		In-Hospital Mortality		One-Year Mortality	
Symptom 1	Symptom 2	No.	*n*	OR (95% CI)	*n*	OR (95% CI)	*n*	OR (95% CI)	*n*	OR (95% CI)
Weakness	Fatigue	275	120	1.48 (1.09–2) *	22	1.29 (0.78–2.06)	9	2.45 (1.06–5.12) *	21	1.29 (0.74–2.13)
Weakness	Dizziness	200	71	0.91 (0.64–1.29)	9	0.65 (0.3–1.25)	5	2.04 (0.68–4.97)	12	1.13 (0.56–2.09)
Weakness	Headache	184	62	1.13 (0.77–1.64)	18	2.03 (1.15–3.41) **	5	3.01 (0.99–7.54) *	8	1.1 (0.47–2.27)
Weakness	Nausea	152	51	0.99 (0.66–1.48)	4	0.42 (0.13–1.02)	2	1.36 (0.22–4.67)	9	1.49 (0.67–3)
Weakness	Loss of appetite	124	57	1.88 (1.21–2.92) **	7	0.9 (0.37–1.87)	1	0.58 (0.03–2.85)	6	0.77 (0.26–1.84)
Weakness	Cough	121	55	1.61 (1.03–2.51) *	8	0.99 (0.43–1.98)	2	1.22 (0.19–4.2)	7	1.09 (0.44–2.35)
Weakness	Fever	121	56	1.91 (1.22–2.99) **	6	0.74 (0.28–1.61)	0	-	6	0.84 (0.31–1.91)
Cough	Fever	160	74	2.14 (1.44–3.17) ***	9	0.9 (0.41–1.74)	0	-	8	0.87 (0.37–1.8)
Headache	Fever	147	52	1.74 (1.15–2.63) **	5	0.69 (0.24–1.57)	0	-	1	0.2 (0.01–0.93)
Headache	Dizziness	281	86	0.97 (0.71–1.33)	12	0.77 (0.4–1.36)	5	1.98 (0.66–4.84)	9	0.76 (0.35–1.5)
Headache	Cough	163	56	1.36 (0.91–2.03)	9	1.06 (0.49–2.06)	2	1.58 (0.25–5.54)	3	0.57 (0.14–1.6)
Headache	Fatigue	162	49	1.06 (0.71–1.59)	14	1.98 (1.05–3.51) *	3	2.65 (0.61–8)	5	0.96 (0.33–2.28)
Headache	Nausea	153	39	0.83 (0.54–1.26)	1	0.12 (0.01–0.54) *	1	1.23 (0.07–6.16)	5	1.55 (0.52–3.72)
Fatigue	Dizziness	158	64	1.29 (0.87–1.91)	10	0.97 (0.46–1.82)	5	2.59 (0.85–6.38)	12	1.53 (0.75–2.9)
Dyspnoea	Chest pain	156	79	1.08 (0.73–1.6)	12	0.68 (0.35–1.22)	5	1.26 (0.42–3.05)	17	1.51 (0.82–2.66)
Dyspnoea	Cough	126	81	2.14 (1.39–3.35) ***	10	0.73 (0.35–1.37)	4	1.22 (0.35–3.19)	14	1.37 (0.71–2.48)
Nausea	Abdominal pain	152	53	1.27 (0.85–1.88)	4	0.41 (0.12–1.01)	2	1.48 (0.23–5.25)	6	1.16 (0.43–2.61)
Nausea	Vomiting	182	67	1.39 (0.96–2)	7	0.63 (0.26–1.32)	2	0.96 (0.15–3.41)	5	0.7 (0.24–1.63)
Nausea	Dizziness	200	58	0.89 (0.62–1.28)	4	0.35 (0.11–0.84) *	2	1.31 (0.21–4.54)	8	1.23 (0.53–2.52)
Leg pain	Joint pain	167	57	1.7 (1.13–2.55) *	8	1.05 (0.44–2.19)	0	-	4	0.48 (0.14–1.2)

No. = Prevalence of the combination of symptom 1 and symptom 2 in the study population; OR = Odds ratio; CI = Confidence interval; * *p* < 0.05, ** *p* < 0.01, *** *p* < 0.001.

## Appendix A

**Table A1:** Distribution of the 5 most frequent combinations of three symptoms.

Combination of Symptoms			Prevalence		Age	Male Sex	No. of Symptoms	ESI
Symptom 1	Symptom 2	Symptom 3	*n*	(%)	Median (year)	(%)	Median	Median
Fatigue	Weakness	Dizziness	106	(2.7)	53	34.0	7	3
Headache	Fatigue	Weakness	104	(2.6)	45.5	36.5	8	3
Headache	Weakness	Dizziness	102	(2.6)	43.5	34.3	7	3
Headache	Dizziness	Nausea	99	(2.5)	44	32.3	6	3
Headache	Fatigue	Dizziness	94	(2.4)	42	37.2	8	3

ESI = Emergency Severity Index.

**Table A2:** Distribution of the 3 most frequent combinations of four symptoms.

Combination of Symptoms				Prevalence		Age	Male Sex	No. of Symptoms	ESI
Symptom 1	Symptom 2	Symptom 3	Symptom 4	*n*	(%)	Median (year)	(%)	Median	Median
Headache	Fatigue	Weakness	Dizziness	62	(1.6)	43.5	35.5	9	3
Loss of appetite	Headache	Fatigue	Weakness	51	(1.3)	42	33.3	10	3
Loss of appetite	Fatigue	Weakness	Dizziness	50	(1.3)	52	30.0	9	3

ESI = Emergency Severity Index.

## Appendix B

**Table A3:** Logistic regression analysis adjusted for age and sex. For detailed discussion see Bingisser et al. [3].

Symptom	No.	Hospitalisation		ICU Admission		In-Hospital Mortality		1-Year Mortality	
		*n*	OR (95% CI)	*n*	OR (95% CI)	*n*	OR (95% CI)	*n*	OR (95% CI)
Headache	707	190	0.96 (0.78–1.17)	38	1.16 (0.8–1.66)	7	0.99 (0.4–2.09)	21	0.71 (0.43–1.13)
Leg pain	627	200	1.05 (0.85–1.28)	24	0.64 (0.41–0.97) *	4	0.42 (0.12–1.03)	20	0.52 (0.31–0.84) **
Dizziness	609	214	1.26 (1.02–1.54) *	29	0.83 (0.54–1.22)	10	1.24 (0.58–2.4)	33	1.01 (0.66–1.50)
Weakness	556	243	1.77 (1.44–2.18) ****	48	1.63 (1.15–2.28) **	15	1.89 (0.99–3.42) *	49	1.56 (1.07–2.22) *
Back pain	510	151	0.88 (0.70–1.10)	14	0.44 (0.24–0.74) **	2	0.25 (0.04–0.82)	21	0.74 (0.44–1.17)
Arm pain	498	127	0.81 (0.64–1.02)	25	1.00 (0.63–1.51)	1	0.17 (0.09–0.76)	12	0.51 (0.26–0.91) *
Abdominal pain	480	170	1.5 (1.19–1.88) ***	19	0.73 (0.44–1.16)	6	1.08 (0.41–2.38)	26	1.23 (0.77–1.91)
Nausea	468	157	1.4 (1.11–1.76) **	17	0.71 (0.41–1.15)	4	0.86 (0.26–2.18)	18	1.03 (0.60–1.70)
Fatigue	452	182	1.68 (1.33–2.10) ****	33	1.41 (0.94–2.06)	10	1.69 (0.79–3.31)	33	1.46 (0.94–2.19)
Chest pain	450	181	1.43 (1.14–1.79) **	45	1.94 (1.36–2.73) ***	9	1.33 (0.6–2.65)	32	1.22 (0.79–1.83)
Cough	417	187	1.9 (1.51–2.39) ****	21	0.82 (0.5–1.28)	6	0.92 (0.35–2.02)	27	1.07 (0.67–1.64)
Dyspnoea	396	233	2.99 (2.36–3.80) ****	36	1.42 (0.96–2.06)	13	1.77 (0.9–3.29)	55	2.31 (1.61–3.28) ****
Joint pain	396	106	0.82 (0.63–1.06)	12	0.54 (0.28–0.94) *	1	0.18 (0.01–0.84)	16	0.82 (0.46–1.39)
Fever	357	170	2.8 (2.18–3.59) ****	18	0.93 (0.55–1.49)	1	0.21 (0.01–0.96)	19	1.12 (0.65–1.85)
Vomiting	229	89	1.94 (1.42–2.66) ****	11	0.99 (0.5–1.78)	3	1.23 (0.29–3.49)	11	1.27 (0.62–2.40)
Flank pain	205	73	1.23 (0.88–1.71)	12	1.07 (0.55–1.88)	2	0.70 (0.11–2.34)	8	0.74 (0.32–1.50)
Loss of appetite	203	93	2.27 (1.64–3.13) ****	8	0.70 (0.31–1.35)	2	0.73 (0.12–2.41)	12	1.16 (0.58–2.13)
Neck pain	191	30	0.48 (0.31–0.72) ***	4	0.44 (0.13–1.05)	0	–	5	0.84 (0.29–1.97)
Sleeping disorder	160	55	1.11 (0.76–1.60)	4	0.40 (0.12–0.97)	4	1.82 (0.53–4.71)	9	0.95 (0.42–1.90)
Gait disorder	154	84	2.01 (1.40–2.90) ***	19	1.93 (1.13–3.15) *	4	1.16 (0.34–2.96)	13	0.91 (0.47–1.65)
Diarrhoea	153	68	1.89 (1.31–2.73) ***	4	0.43 (0.13–1.04)	2	0.95 (0.15–3.18)	11	1.44 (0.69–2.73)
Dysphagia	146	35	0.93 (0.61–1.41)	6	0.93 (0.36–1.98)	0	–	4	0.87 (0.25–2.28)
Nasal discharge	140	33	0.76 (0.48–1.16)	0	–	1	0.71 (0.04–3.36)	3	0.49 (0.12–1.39)
Numbness	140	56	1.88 (1.27–2.76) **	19	3.19 (1.85–5.24) ****	1	0.64 (0.04–3.04)	5	0.78 (0.27–1.85)
Expectoration	122	61	2.04 (1.36–3.06) ***	5	0.62 (0.22–1.39)	2	0.95 (0.15–3.2)	9	1.10 (0.49–2.19)
Dysuria	112	25	0.44 (0.26–0.71) **	1	0.13 (0.007–0.57) *	1	0.44 (0.02–2.09)	7	0.75 (0.30–1.63)
Joint swelling	108	35	0.98 (0.62–1.52)	3	0.46 (0.11–1.24)	1	0.61 (0.03–2.95)	7	1.20 (0.47–2.64)
Altered state of mind	105	44	1.36 (0.87–2.11)	8	1.27 (0.56–2.51)	1	0.58 (0.03–2.74)	5	0.74 (0.25–1.74)
Leg swelling	82	34	1.28 (0.78–2.11)	2	0.35 (0.06–1.12)	0	–	4	0.57 (0.16–1.49)
Obstipation	80	31	1.08 (0.65–1.77)	2	0.37 (0.06–1.2)	2	1.63 (0.26–5.57)	7	1.47 (0.58–3.20)
Acute visual disorder	68	21	1.24 (0.68–2.19)	3	0.89 (0.21–2.46)	1	1.35 (0.07–6.75)	3	1.03 (0.23–3.17)
Skin rash	66	16	1.23 (0.65–2.22)	1	0.39 (0.02–1.8)	0	–	1	0.81 (0.04–4.07)
Paralysis	53	31	2.84 (1.54–5.31) ***	15	6.38 (3.3–11.82) ****	2	2.29 (0.36–8.02)	6	1.88 (0.68–4.46)
Speech disorder	46	33	4.95 (2.46–10.51) ****	15	7.15 (3.62–13.57) ****	2	2.16 (0.34–7.6)	6	1.66 (0.59–4.02)
Acute hearing disorder	33	6	0.68 (0.24–1.65)	1	0.73 (0.04–3.5)	0	–	0	–

No. = Prevalence of the symptom in the study population; OR = Odds ratio; CI = Confidence interval; * *p* < 0.05, ** *p* < 0.01, *** *p* < 0.001, **** *p* < 0.0001.
